# Gaining new understanding of sarcomere length non-uniformities in skeletal muscles

**DOI:** 10.3389/fphys.2023.1242177

**Published:** 2024-01-11

**Authors:** Meng Li, T. R. Leonard, S. W. Han, E. K. Moo, W. Herzog

**Affiliations:** ^1^ Human Performance Lab, University of Calgary, Calgary, AB, Canada; ^2^ Institute of Physiology II, University of Münster, Münster, Germany; ^3^ Department of Technical Physics, University of Eastern Finland, Kuopio, Finland; ^4^ Department of Mechanical and Aerospace Engineering, Carleton University, Ottawa, ON, Canada

**Keywords:** sarcomere length non-uniformity, sarcomere contraction dynamics, passive structures, cross-bridges, myofibrils, skeletal muscle properties

## Abstract

Sarcomere lengths are non-uniform on all structural levels of mammalian skeletal muscle. These non-uniformities have been associated with a variety of mechanical properties, including residual force enhancement and depression, creep, increased force capacity, and extension of the plateau of the force-length relationship. However, the nature of sarcomere length non-uniformities has not been explored systematically. The purpose of this study was to determine the properties of sarcomere length non-uniformities in active and passive muscle. Single myofibrils of rabbit psoas (*n* = 20; with 412 individual sarcomeres) were subjected to three activation/deactivation cycles and individual sarcomere lengths were measured at 4 passive and 3 active points during the activation/deactivation cycles. The myofibrils were divided into three groups based on their initial average sarcomere lengths: short, intermediate, and long average sarcomere lengths of 2.7, 3.2, and 3.6 µm. The primary results were that sarcomere length non-uniformities did not occur randomly but were governed by some structural and/or contractile properties of the sarcomeres and that sarcomere length non-uniformities increased when myofibrils went from the passive to the active state. We propose that the mechanisms that govern the systematic sarcomere lengths non-uniformities observed in active and passive myofibrils may be associated with the variable number of contractile proteins and the variable number and the adjustable stiffness of titin filaments in individual sarcomeres.

## 1 Introduction

It has been well acknowledged that sarcomeres in vertebrate muscles are non-uniform in length. Huxley and Peachey (Huxley and Peachey, 1961) measured sarcomere lengths in passive frog semitendinosus and iliofibularis muscles and found that sarcomeres towards the ends of the fibres were much shorter (1.8 and 2.7 µm for semitendinosus and iliofibularis, respectively), compared to sarcomeres in the centre of the corresponding fibres (4.2 and 3.8 µm for semitendinosus and iliofibularis, respectively). Similar results have been reported for *in vivo* human and mouse muscles (Llewellyn et al., 2008; Moo et al., 2016; Moo and Herzog, 2018) and in single myofibrils from rabbit psoas muscles (Rassier et al., 2003; Johnston et al., 2016; Johnston et al., 2019). Sarcomere length non-uniformities in excess of 1.0 µm were also reported in a recent study on the effects of eccentric training in human biceps femoris muscles using micro-endoscopy and three-dimensional ultrasonography (Pincheira et al., 2021). Furthermore, median sarcomere lengths before and after the exercise interventions in the biceps femoris reported in the study by Pincheira et al. (Pincheira et al., 2021) ranged from about 2.5 to 4.0 µm across their subjects (Herzog and Fontana, 2022), a difference that accounts for approximately half of the theoretical excursion of human skeletal muscle sarcomeres (Walker and Schrodt, 1974; Herzog et al., 1992). Sarcomere lengths non-uniformities have not only been observed in passive (e.g., Huxley and Peachey, 1961; Llewellyn et al., 2008; Johnston et al., 2016; Pincheira et al., 2021) but also in active muscles (Moo et al., 2017) and in fully activated single myofibrils during isometric (Johnston et al., 2016) and after eccentric contractions (Joumaa et al., 2008a; Johnston et al., 2019).

Sarcomere length non-uniformities have been associated with many important mechanical properties of skeletal muscles. For example, the residual force enhancement property of skeletal muscles (Abbott and Aubert, 1952; Edman et al., 1982; Herzog and Leonard, 2002) has been thought to be caused by the development of sarcomere length non-uniformities when an active muscle is stretched, (e.g., Huxley, 1980; Morgan, 1990; Morgan, 1994; Edman and Tsuchiya, 1996; Morgan et al., 2000; Rassier and Pavlov, 2012). Similarly, residual force depression, (e.g., Maréchal and Plaghki, 1979; Herzog and Leonard, 1997) has been thought to be caused by the development of sarcomere length non-uniformities when an active muscle is shortened (e.g., Morgan et al., 2000). Skeletal muscle creep (e.g., Gordon et al., 1966; Edman and Reggiani, 1984) and associated extension of the plateau of the force-length relationship (e.g., Ramsey and Street, 1940; Gordon et al., 1966; ter Keurs et al., 1978) have also been attributed to the development of sarcomere length non-uniformities. This last observation led to the development of equipment systems that allowed, through optical feedback methods, for the control of sarcomere and segment lengths in isometrically contracting muscle fibres (e.g., Gordon et al., 1966; Edman et al., 1982) with the expressed purpose to study muscle fibre segments under truly isometric conditions. Finally, sarcomere length non-uniformities have been thought to cause instability of force production on the descending limb of the force-length relationship (e.g., Hill, 1953; Huxley, 1980) leading to muscle damage characterized by overstretched sarcomeres (e.g., Morgan, 1990; Morgan, 1994; Denoth et al., 2002; Morgan and Proske, 2004; Campbell et al., 2011; Herzog, 2014; Herzog, 2018; Lieber, 2018).

Despite the generally accepted observation that vertebrate skeletal muscles have large sarcomere length non-uniformities in the passive and active state, and the functional importance associated with these sarcomere length non-uniformities, the causes underlying sarcomere length non-uniformities remain unexplored. Even some of the most fundamental questions, such as “do sarcomere length non-uniformities occur randomly or are they deterministic and caused by some structural or contractile properties of the muscle” or “are sarcomere length non-uniformities in the passive and active muscle the same, or are they related to each other in some manner” remain unanswered.

The purpose of this study was to systematically explore the properties of sarcomere length non-uniformities in active (activated) and passive (relaxed) muscles. Specifically, we asked the questions: i) do sarcomere length non-uniformities evolve randomly every time a muscle is activated or deactivated, ii) are sarcomere length non-uniformities the same, or at least similar, in the active and passive muscle, and iii) if not random, what structural or contractile properties might determine sarcomere length non-uniformities? We chose to explore these questions in single myofibrils because this preparation allows for the most accurate determination of sarcomere lengths and guarantees that force transmission across all sarcomeres is identical as all sarcomeres are arranged strictly in series (Herzog and Fontana, 2022). Randomness (or not) of the evolution of sarcomere length non-uniformities was determined by three repeat activation and deactivation cycles at each of three distinct average sarcomere lengths. Different average sarcomere lengths were used to explore the evolution of sarcomere length non-uniformities in the presence of no (or little) passive force (average sarcomere length of about 2.7 µm), with some passive force that was smaller than the active force (about 3.2 µm) and at a length where passive force is about equal or even slightly greater than the active force (about 3.6 µm). We hypothesized that sarcomere length non-uniformities evolve in a predictable way, and thus, that sarcomere lengths remain similar for repeat activation and deactivation cycles in the passive and in the active state. We further hypothesized that sarcomere length non-uniformities increase from the passive to the active state.

## 2 Materials and methods

All materials and methods for the mechanical testing of single myofibrils have been used in our lab before and have been described in detail (e.g., Rassier et al., 2003; Joumaa et al., 2008b; Leonard and Herzog, 2010). They are briefly summarized below.

### 2.1 Specimen

Strips of rabbit psoas muscle were taken from euthanized animals and tied to wooden sticks to preserve the *in situ* sarcomere length. These strips were then placed in a rigor-glycerol solution with protease inhibitors (Complete, Roche Diagnostics, Montreal, QB, Canada) and stored at −20°C for 10–14 days. On the day of experimentation, strips of muscle were placed in a 4°C rigor solution, homogenized, and placed in the experimental chamber (20°C). The solutions (rigor, activation and relaxation solutions) have been described before (e.g., Tesi et al., 2002; Rassier et al., 2003; Han et al., 2023). Ethics approval was granted from the institutional Animal Ethics Committee at the University of Calgary.

The experimental chamber was comprised of a conical Teflon-lined container with a glass coverslip on the bottom, and the entire assembly placed on top of an inverted microscope (Zeiss, Axiovert 200M, Germany). After a sufficient time for stabilization (about 10 min), the rigor solution was replaced with a relaxing solution, and myofibrils in suspension were washed away leaving those attached to the coverslip of the experimental chamber. A myofibril with a good striation pattern was then selected and attached to a glass needle and motor at one end, and to a reinforced pair of nano-levers at the other end to reduce compliance of the system (e.g., Leonard and Herzog, 2010).

### 2.2 Protocol and sarcomere length measurements

**FIGURE 1:**
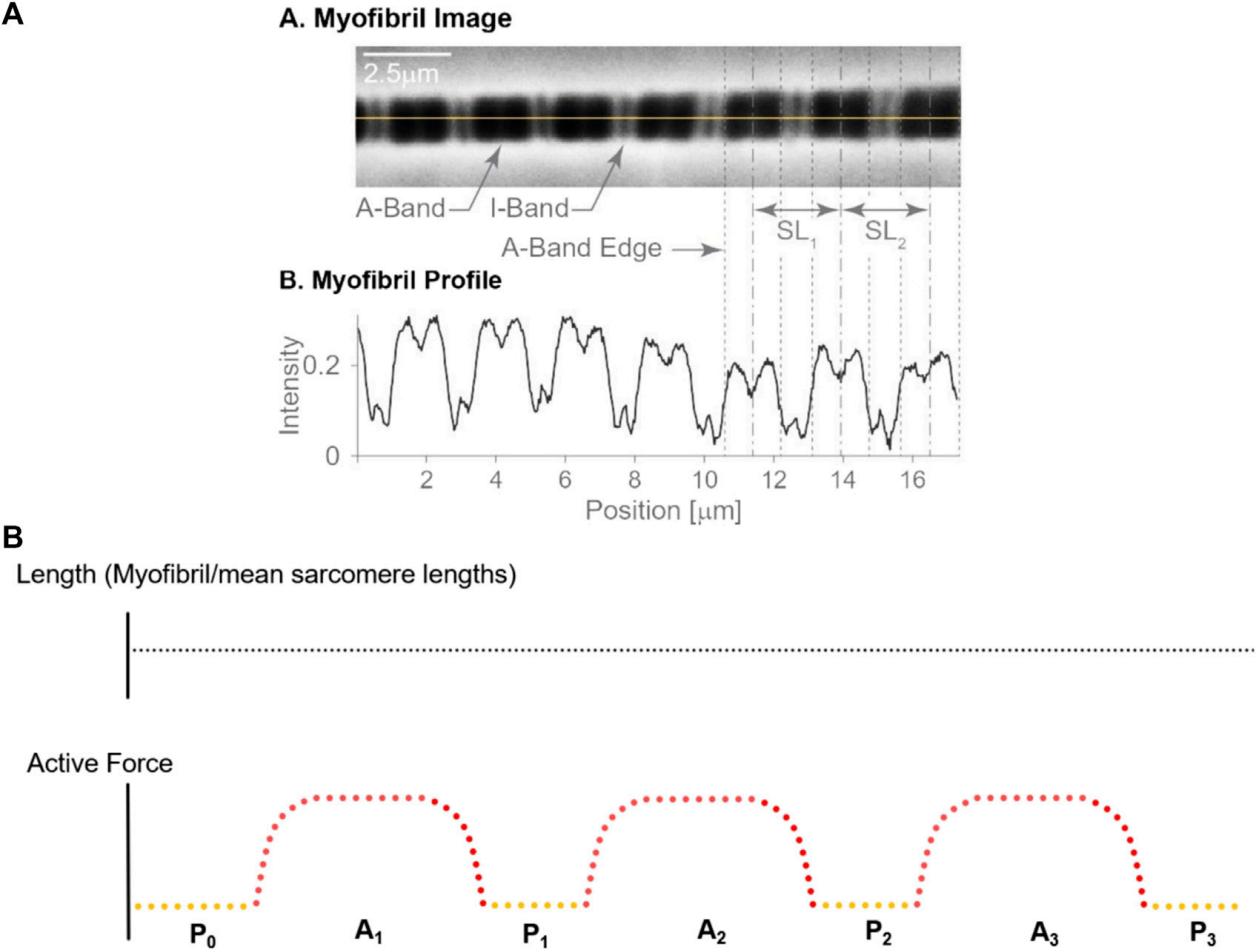
**(A)** Sarcomere length measurement: An isolated single myofibril was readied for mechanical assessment **(A)**, along with the corresponding dark-light intensity patterns caused by sarcomere structures. The dark A-bands and Z-lines show high intensity, while the light I-bands show a low intensity **(B)**. The loss of intensity in the middle of the A-band indicates the H-zone, that is the region in the centre of the A-band that does not contain actin filaments. The length of a sarcomere was measured by the distance of adjacent A-band centroids, the location of which is indicated by the dashed vertical lines, see SL1 and SL2. Adapted from Schmidt et al. (2021); with permission from Elsevier. **(B)** Experimental Protocol: Sarcomere lengths were measured at seven different instances during the experimental protocol that involved three activation-deactivation cycles. The total myofibril length remained unchanged throughout these cycles. Yellow dots indicate the relaxed states with sarcomere length measurements made prior to activation (P0) and following the three activation-deactivation cycles (P1, P2 and P3, respectively); Red dots indicate the active states with measurements made during the three activation periods (A1, A2, and A3, respectively), once steady state forces were reached.

20 myofibrils, from 8 female rabbits, with a total number of 412 sarcomeres underwent three isometric activation-deactivation cycles at nominal average sarcomere lengths of 2.7 µm (short group; negligible passive force), 3.2 µm (middle group; passive force < active force), and 3.6 µm (long group; passive force ≥ active force) (Figure 1B). There were 156 sarcomeres in the short group, 121 sarcomeres in the middle group and 135 sarcomeres in the long group. Sarcomere lengths within a myofibril were measured at 7 distinct steady states (Figure 1B): (i) before the first activation cycle (passive, referred to as P_0_), and (ii) in the first passive state after the first, (iii) second and (iv) third activation cycle (hereafter referred to as P_1_, P_2_, and P_3_, respectively), for four measurements of individual sarcomere length in the passive state. Sarcomere lengths were also measured once the maximal steady state forces were reached in the active myofibrils for the (v) first activation cycle, the (vi) second and (vii) the third activation cycle (hereafter referred to as A_1_, A_2_, A_3_) for a total of three sarcomere length measurements in the active myofibrils (see video1 for activation and video 2 for deactivation in the supplementary material). All specimens were maximally activated using a pCa [4.0] (Bartoo et al., 1993).

The lengths of individual sarcomeres were measured using the intensity profiles created by the I-bands (light intensity) and A-bands (dark intensity) (e.g., Johnston et al., 2016; Johnston et al., 2019; Schmidt et al., 2021). I-bands and A-bands were defined by appropriate intensity thresholds and were demarcated for each sarcomere. Once demarcated, the centroid of the A-bands was determined through an area integration, and sarcomere lengths were calculated from one A-band centroid to the adjacent A-band centroids (Figure 1A). Sarcomere lengths were measured using custom written software (Schmidt et al., 2021). Sarcomere lengths of selected myofibrils obtained in a single experiment were measured three times on three different days to test the intra-tester reliability of the sarcomere length measurements.

Sarcomere length changes between the passive and the activated state were obtained by subtracting the sarcomere length in the passive state from that of the active state. Therefore, a negative number indicates a shortening of a sarcomere during activation and a positive number indicates a lengthening.

### 2.3 Reliability of sarcomere length measurements

Reliability depends on several factors: the chosen threshold for distinguishing between I- and A-bands, the selection of the specific line segment along the myofibril that is chosen for the sarcomere length measurement, and, in situations where the automated system fails to detect A-band edges, the subjective assessment of the researcher. To determine the reliability of the sarcomere length measurements, sarcomere lengths of nine myofibrils and 209 sarcomeres were measured on three separate occasions spaced at least 3 days apart.

### 2.4 Statistical analysis

Sarcomere length non-uniformities were quantified by calculating the standard deviations (SD) and coefficients of variation (CoV) for each group of sarcomeres (short, medium, and long average sarcomere lengths). To determine if sarcomere lengths in the active and passive states during repeat activation-deactivation cycles occurred randomly or were related, individual sarcomere lengths for P_0_ and P_1_, P_0_ and P_2_, P_0_ and P_3_, A_1_ and A_2_, and A_1_ and A_3_ were plotted against each other and fitted using a best fitting linear regression approach that was forced through the origin. To determine if, and what kind of relationship there may exist between sarcomere lengths in the passive and the active states, linear approximations between sarcomere lengths P_0_ and A_1_, P_1_ and A_2_, and P_2_ and A_3_ were quantified. A level of significance of α = 0.05 was adopted *a priori*.

## 3 Results

When quantifying the same sarcomere lengths on three different occasions, 95% of all repeat measurements were within 0.2 µm from the original (first measurement) value. Therefore, we adopted 0.2 µm as the intra-examiner reliability of our sarcomere length measurement, thus all sarcomere lengths falling within 0.2 µm of a “reference” value were considered the same.

**FIGURE 2:**
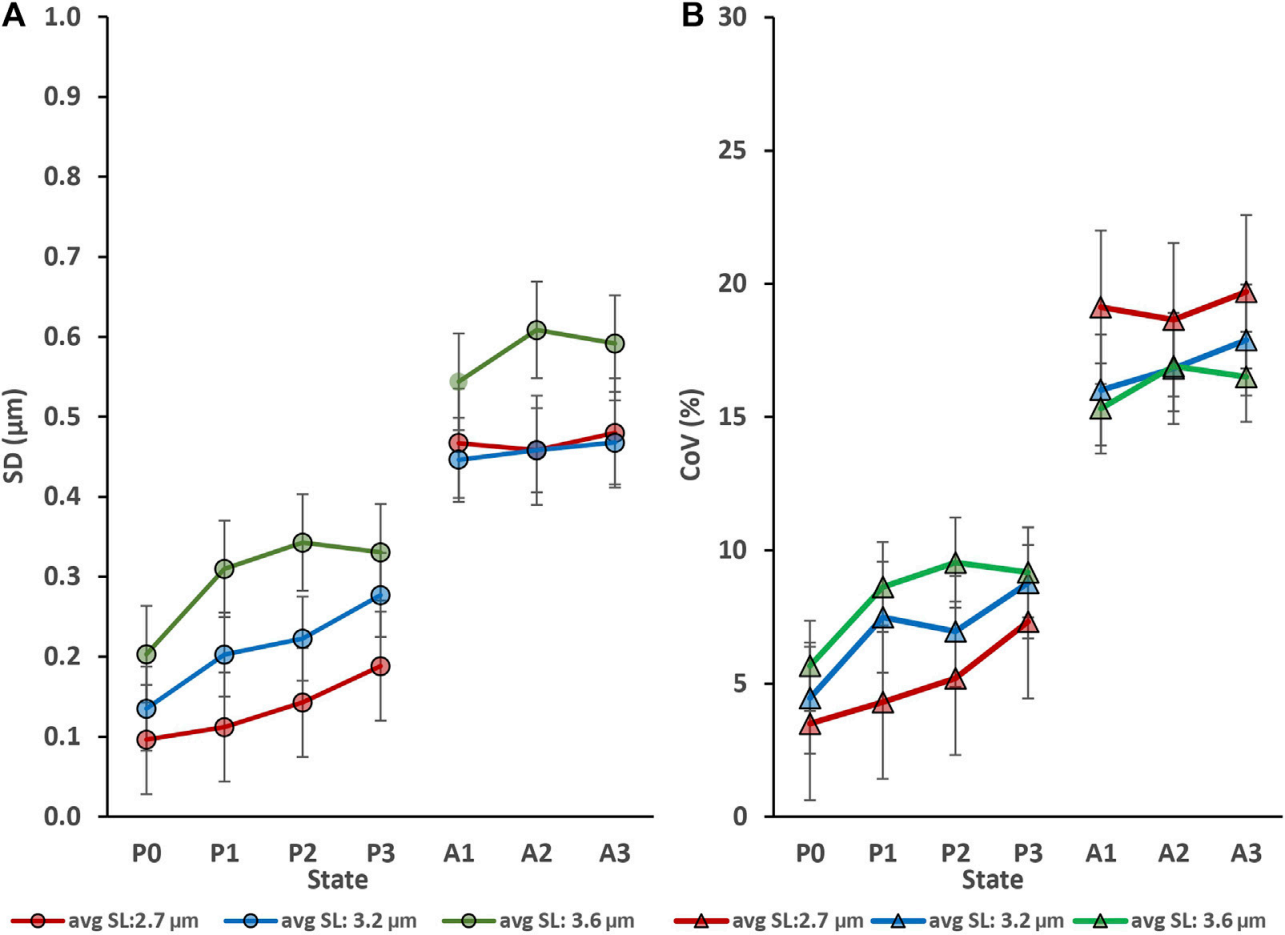
Activation (going from the relaxed to the activated state) produced an increase in sarcomere lengths non-uniformities at each average sarcomere length that was tested but repeat activation/deactivation did not change sarcomere length non-uniformity in the passive conditions (P0 to P3) or the active conditions (A1 to A3). **(A)** Sarcomere length non-uniformity expressed as mean standard deviations expressed in µm. **(B)** Sarcomere length non-uniformity expressed as mean coefficients of variation expressed in percent. The absolute sarcomere length non-uniformities were greatest for the longest average sarcomere lengths (3.6 µm) tested **(A)**, but the coefficients of variation were greatest for the shortest (2.7 µm) test group of activated myofibrils **(B)**.

Myofibril activation produced an increase in sarcomere length non-uniformities at each lengths tested (Figure 2); that is, sarcomere length non-uniformities were always greater in the activated compared to the corresponding passive myofibril (*p* < 0.05).

**FIGURE 3:**
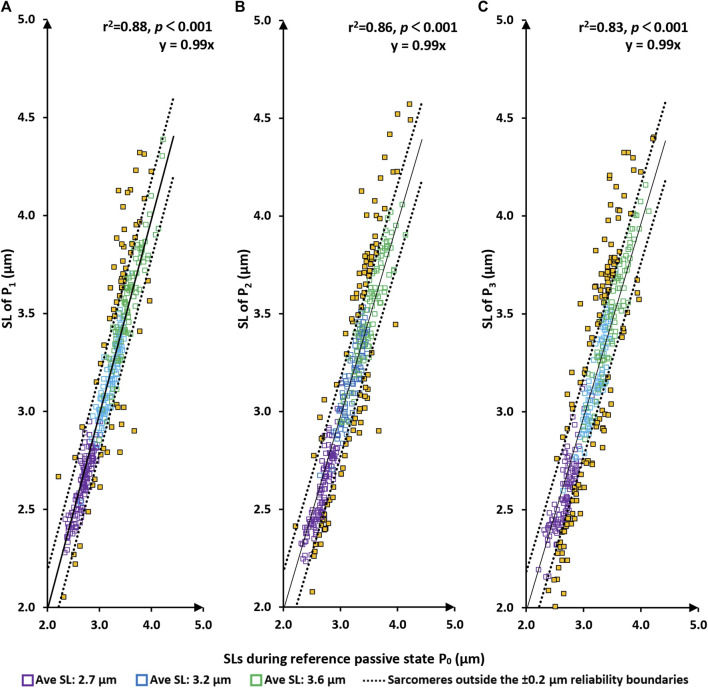
Sarcomere lengths of the original passive state (P0 = before the first activation) vs. sarcomere lengths in the passive/relaxed state after the first, second and third activation/deactivation cycles (P1–panel **(A)**; P2–panel **(B)**; and P3–panel **(C)**, respectively). Red squares are sarcomeres from the short group (nominal average sarcomere lengths of 2.7 µm). Blue squares are from the middle group (nominal average sarcomere length of 3.2 µm). Green squares are from the long group (nominal average sarcomere length of 3.6 µm). Yellow squares are sarcomeres whose length changes are greater than the reliability of measurement off 0.2 µm. The solid line represents the best fitting linear regression that is forced through the origin (0/0) of the coordinate system. The dotted lines are offset from the best fitting regression line by ±0.2 µm, which represents the day-to-day reliability of sarcomere length measurements. In other words, sarcomeres within these lines are considered to be of the same length. *p* < 0.001, there is a very statistically significant relationship between the variables.

There was a tight linear relationship between the sarcomere lengths measured at the P_0_ (the initial passive state) and those measured at P_1_, P_2_, and P_3_ (Figure 3A). Best fitting linear regressions forced through the origin had slopes of nearly 1.0 (>0.99 in each case) and coefficients of determination, r^2^, of 0.88, 0.86, and 0.83, respectively with *p*-values in all cases *p* < 0.001.

**FIGURE 4:**
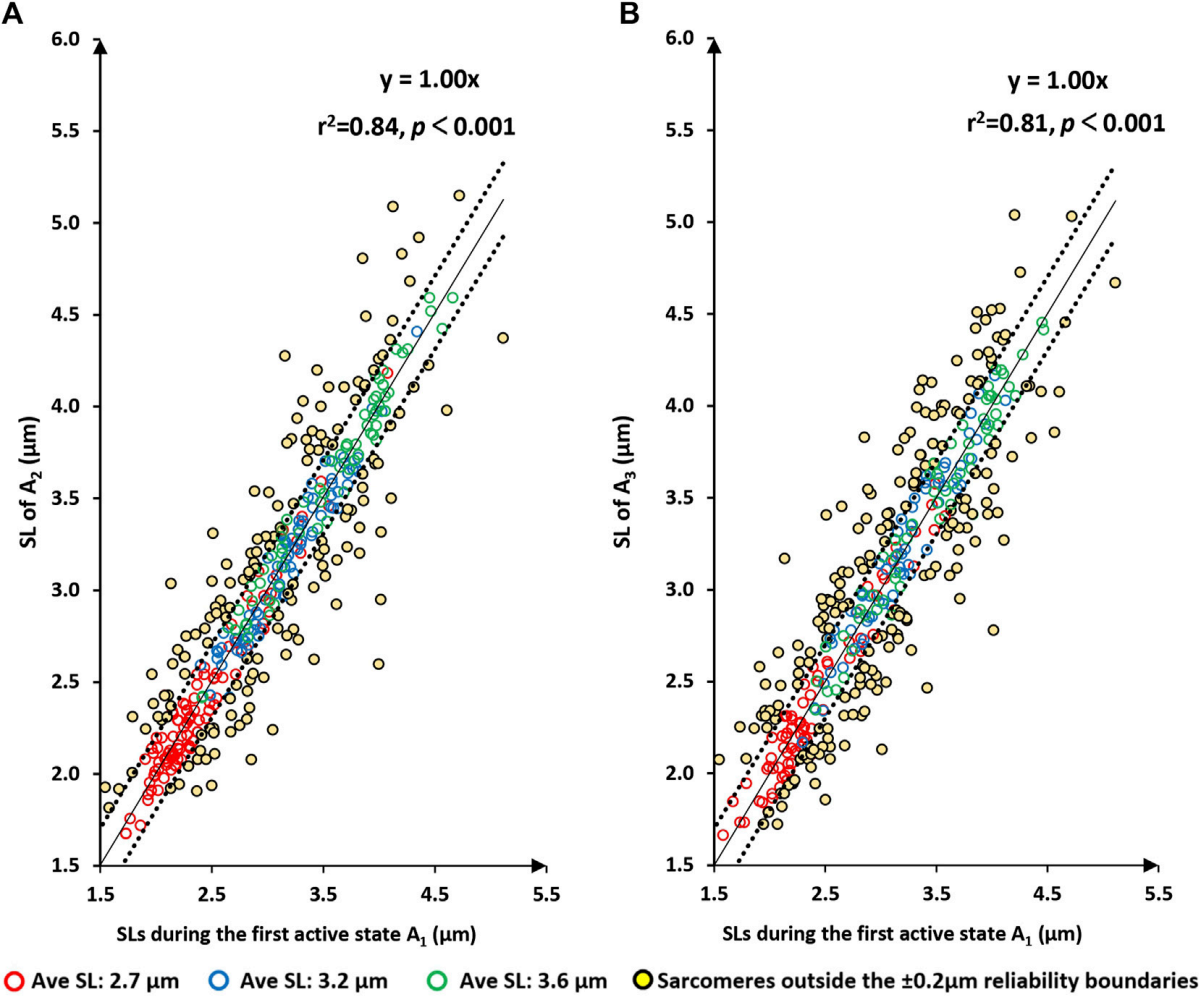
Sarcomere lengths for the first active state (A1 = first activation) vs. sarcomere lengths in the active state following the second and third activation/deactivation cycles (A2 – panel **(A)**; A3 – panel **(B)**, respectively). Red circles are sarcomeres from the short group (nominal average sarcomere length of 2.7 µm). Blue circles are from the middle group (nominal average sarcomere length of 3.2 µm). Green circles are from the long group (nominal average sarcomere length of 3.6 µm). Yellow circles are sarcomeres whose length changes were greater than the day-to-day reliability of ±0.2 µm. The solid line represents the best fitting linear regression that was forced through the origin (0/0) of the coordinate system. The dotted lines are offset from the best fitting regression line by ±0.2 µm, which represents the day-to-day reliability of sarcomere length measurements. Sarcomeres within these dotted lines are considered to be of the same length. *p* < 0.001, there is a very statistically significant relationship between the variables.

Similarly, there was a high linear relationship between the sarcomere lengths measured at the A_1_-state (first active state) and those measured at A_2_ and A_3_ (Figure 4). Best fitting linear regressions forced through the origin had slopes close to 1.0 (1.0026 and 1.0003, respectively) and coefficients of determination, r^2^, of 0.84 and 0.81, respectively with *p*-values in both cases *p* < 0.001.

**FIGURE 5:**
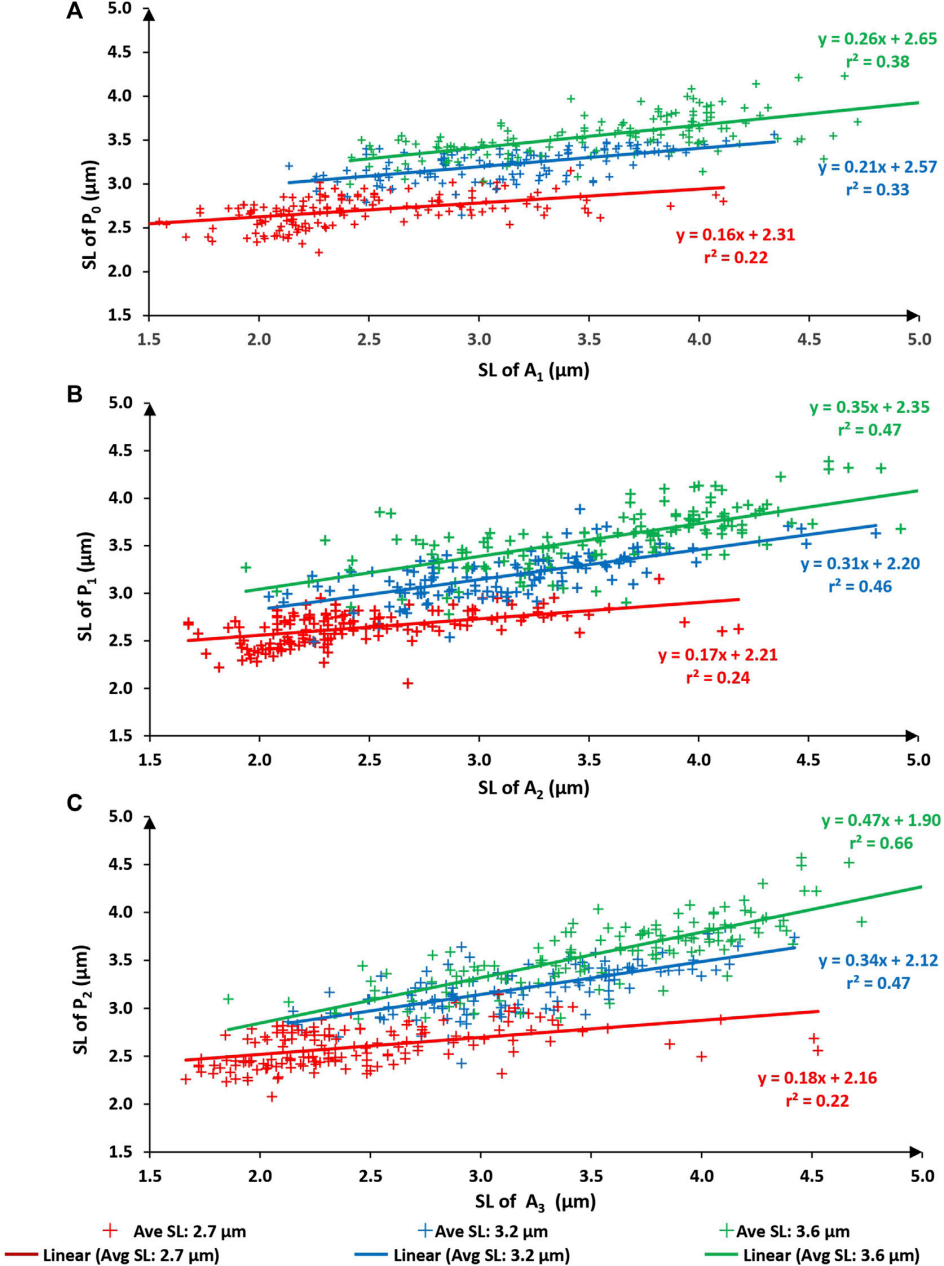
Sarcomere lengths of the first active state (A1) vs. sarcomere lengths of the initial passive state (P0–panel **(A)**. Sarcomere lengths of the second active state (A2) vs. sarcomere lengths in the passive state after the first activation (P1–panel **(B)**. Sarcomere lengths of the third active state (A3) vs. sarcomere lengths in the passive state after the second activation (P2–panel **(C)**). Red symbols are sarcomeres from the short group (nominal average sarcomere lengths of 2.7 µm). Blue symbols are from the middle group (nominal average sarcomere length of 3.2 µm). Green symbols are from the long group (nominal average sarcomere length of 3.6 µm). The solid lines of red, blue, green represent the best fitting linear regression for the short group, middle group, and long group myofibrils respectively.

When comparing sarcomere lengths from the active states to the passive states (that is A_1_ to P_0_; A_2_ to P_1_; and A_3_ to P_2_) the best fitting linear regression equations always had a slope significantly smaller than 1.0 and a substantial positive y-intercept for all three test lengths (Figure 5; average sarcomere lengths of 2.7, 3.2, and 3.6 µm) indicating that upon activation sarcomeres that were long in the passive state tended to become even longer when activated, and sarcomeres that were short in the passive state tended to become even shorter when activated, thereby producing a somewhat predictable expansion of the sarcomere length non-uniformities in the active compared to the passive state, as captured in Figure 2.

**FIGURE 6:**
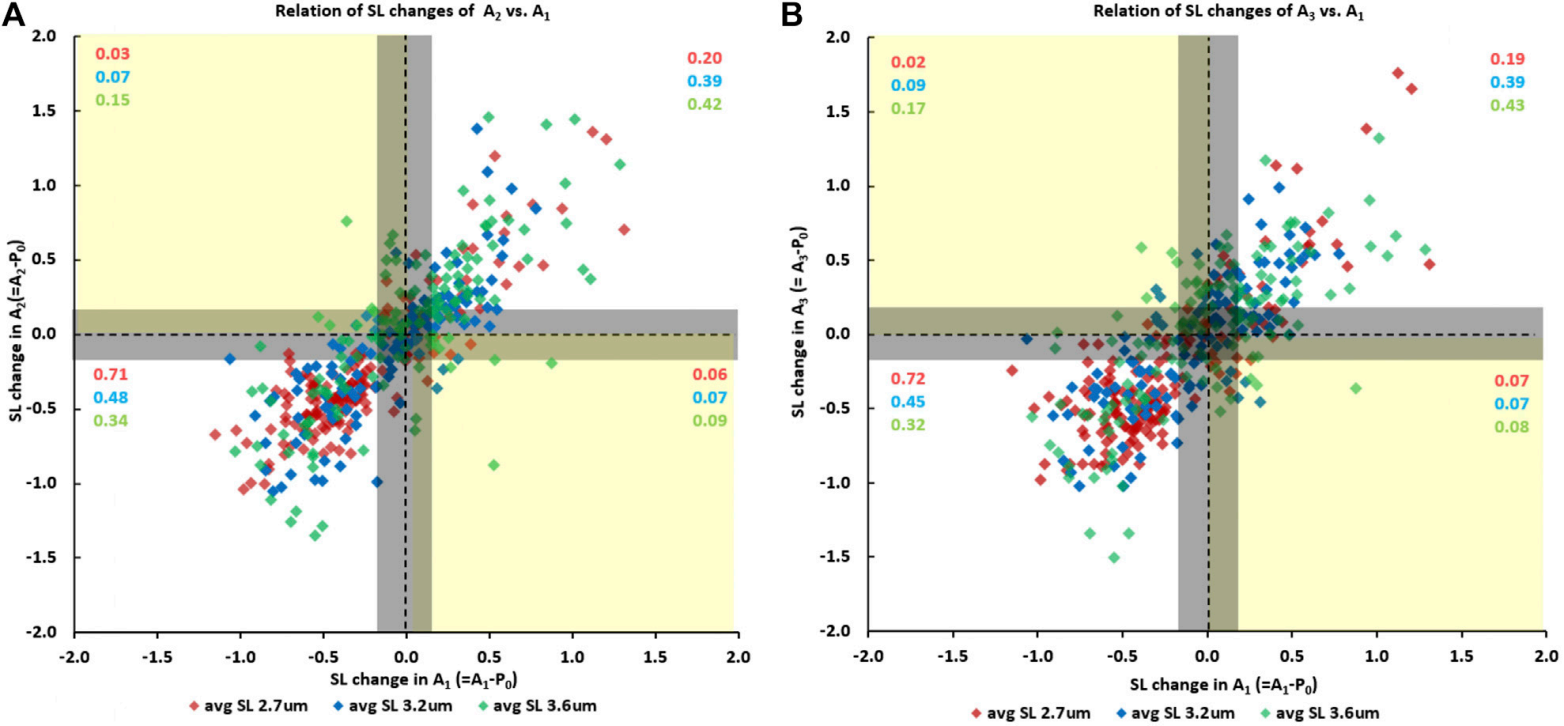
**(A)** Sarcomere length changes in the second activation cycle (going from the initial passive state “P0” to the second active state “A2”) as a function of the sarcomere length changes occurring during the first activation cycle (going from the initial passive state “P0” to the first active state “A1”), and **(B)** the corresponding sarcomere length changes going from the initial passive state “P0” to the third active state “A3” as a function of the length change in the first activation cycle (going from “P0” to “A1”). Data points in the first and third quadrant indicate that sarcomeres were shortening/elongating in both activation cycles, while data points in the second and fourth quadrant indicate that sarcomeres were elongating/shortening or shortening/elongating in the first and subsequent activation cycle. The proportion of sarcomeres falling into the four quadrants are indicated in the red, blue, and green numbers for the short (2.7 µm), middle (3.2 µm) and long (3.6 µm) average sarcomere length tests. The vertical and horizontal lines through the zero values indicate zero length change of a sarcomere in the activation cycle going from the passive to the active state. The corresponding vertical and horizontal lines at ±0.2 µm from the zero lines indicate the reliability of our sarcomere length measurements. In other words, sarcomere length changes within the ± 0.2 µm lines are considered zero length change, or length changes that cannot be said to be greater than zero with a degree if certainty.

Finally, sarcomeres that were shortening when going from the initial passive state (passive state “P_0_”) to the first active state (A_1_) also tended to shorten when myofibrils were activated the second (A_2_) (Figure 6A) and the third time (A_3_) (Figure 6B). Similarly, sarcomeres that elongated when going from the initial passive state to the first active state also tended to elongate when myofibrils were activated for the second (Figure 6A) and the third time (Figure 6B). The proportion of sarcomeres shortening or elongating consistently (indicated by the purple, blue and green numbers in the first and third quadrants of Figure 6A and Figure 6B) was 0.91 for tests at an average sarcomere lengths of 2.7 µm (red numbers in Figure 6A and Figure 6B), was 0.87 and 0.83 for the tests at an average sarcomere length of 3.2 µm, (blue numbers in Figure 6A and Figure 6B), and was 0.76 and 0.74 for the tests at an average sarcomere length of 3.6 µm (green numbers in Figure 6A and Figure 6B). When accounting for the reliability of sarcomere length measurements of 0.2 µm, there only remained a handful of cases in which sarcomeres that were shortening in one activation cycle were lengthening in another or *vice versa*.

## 4 Discussion

The primary results of this study were (i) that sarcomere length non-uniformities did not occur randomly but seemed governed by some structural and/or contractile properties of the sarcomeres (Figure 3A, B), and (ii) that sarcomere length non-uniformities differ greatly between the passive and active state. Specifically, sarcomere length non-uniformities consistently increased from the passive to the active state at all average sarcomere lengths (Figure 2) and for each myofibril tested. However, despite the differences in sarcomere length non-uniformities between passive and activated sarcomeres, there was some predictability about how sarcomere lengths were changing upon activation. Sarcomeres that tended to be short in the passive myofibril tended to remain short upon activation and sarcomeres that tended to be long, tended to remain long (Figure 5). Finally (iii), when sarcomeres shortened when activated the first time, they tended to shorten in subsequent activation cycles, and *vice versa*, if sarcomeres elongated when activated the first time, they tended to do so in subsequent activation cycles as well (Figure 6).

The results of this study confirm two previously published results: first, sarcomere lengths are non-uniform in active and passive muscles. This finding has been made for all structural levels of muscle ranging from whole muscles (Moo et al., 2016; Moo et al., 2017; Moo and Herzog, 2018; Pincheira et al., 2021) to single fibres (Julian and Morgan, 1979; Iwazumi and Pollack, 1981; Edman and Reggiani, 1984), and myofibrils (Bartoo et al., 1997; Johnston et al., 2016; Johnston et al., 2019). Second, sarcomere length non-uniformities increase substantially when a muscle is activated (Figure 2), that is, when a muscle, fibre or myofibril goes from the passive state to an active state. Novel to the literature is the result that sarcomere lengths non-uniformities do not occur randomly, neither in passive nor in active myofibrils, but they appear to be “governed” by some rules or constraints. This interpretation is supported by the tight proportionality of the best fitting linear regression lines (forced through zero) that have slopes close to 1.0 (range from 0.989 to 1.003) and coefficients of determination ranging from 0.81 to 0.88 for measurements of sarcomere lengths obtained repeatedly for the same contractile conditions (Figures 3, 4).

When relating the sarcomere length non-uniformities in the active conditions to the sarcomere length non-uniformities in the passive condition that immediately preceded the active condition, attempting to fit linear regression lines that were forced through zero produced unacceptable results, that is, regression lines, that in contrast to those obtained in Figures (Figures 3, 4), were far from the best fitting regression lines. Furthermore, the coefficients of variation relating passive to the corresponding active sarcomere lengths (Figure 5) were much smaller than those relating sarcomere lengths between repeated passive and repeated active states (Figures 3, 4). This result suggested to us that the sarcomere length non-uniformities in the passive and active state may have been caused by different mechanisms. However, this suggestion will need direct exploration in future studies. Best fitting regression lines between the passive and active state, calculated independently for the three length conditions, gave regression lines with a slope substantially smaller than 1.0 (range from 0.16 to 0.47) and positive Y-value intercepts ranging from 1.90 to 2.65 (Figure 5). A slope of smaller than 1.0 and a positive Y-intercept indicates that when a myofibril was activated, sarcomeres that were short in the passive state tended to become even shorter with activation, and sarcomeres that were long in the passive state tended to become even longer with activation. Sarcomeres that were of medium length tended to stay at about the same length. In fact, for the 9 regression lines in Figure 5, one can calculate the sarcomere length that, on average, remained unchanged from the passive to the active state. These unchanged sarcomere lengths ranged from 3.14 µm to 3.66 µm, with an average of 3.39 µm. These results suggest that sarcomere length non-uniformities in the active state increased because of a dispersion of sarcomeres at the long and short end of sarcomere lengths.

An interesting observation was that the proportion of sarcomeres that shortened or lengthened upon activation depended crucially on the average sarcomere length. For example, for the shortest average sarcomere length (2.7 µm), the proportion of sarcomeres that tended to shorten repeatedly upon myofibril activation was 0.71 and 0.72 for the situations shown in Figure 6A and Figure 6B), while the proportion that lengthened was 0.20 and 0.19, respectively. Since the average sarcomere length remained the same for the passive and activated myofibrils, this result implies that the average distance shortened by the “shortening sarcomeres” was smaller than the average elongation of the “lengthening sarcomeres”, as can be seen qualitatively in Figure 6A and Figure 6B. In contrast, for the longest average sarcomere length (3.6 µm), the situation was reversed; that is the proportion of sarcomeres that tended to shorten repeatedly when going from the passive to the activated state was smaller (0.34 and 0.32, Figure 6A and Figure 6B), than the proportion of sarcomeres that tended to elongate (0.42 and 0.43, respectively).

Furthermore, when going from the active to the passive state, the coefficient of determination increased with increasing average sarcomere lengths. The coefficients of variation at the shortest testing length (average sarcomere length of about 2.7 µm) were 0.22, 0.24, and 0.22 when relating the A_1_-state to P_0_, A_2_ to P_1_, and A_3_ to P_2_, respectively (Figure 5). The corresponding coefficients of determination at the longest myofibril testing conditions were 0.38, 0.46 and 0.66, respectively, indicating that predicting the length of sarcomeres in the active state from the passive state was better when the average sarcomere lengths were long. We propose that this result may be because at the different test lengths, the contribution of passive and active elements to sarcomere length non-uniformities changes systematically. At the shortest test length of about 2.7 µm/sarcomere, passive force in rabbit psoas myofibrils is essentially zero (Horowits, 1992; Bartoo et al., 1997; Leonard and Herzog, 2010), at the intermediate testing length (about 3.2 µm/sarcomere) passive forces reach about 10%–20% of the maximal isometric force achieved at optimal sarcomere length (Bartoo et al., 1993; Bartoo et al., 1997; Rassier et al., 2003), and finally, at the longest testing length (about 3.6 µm/sarcomere), the passive forces in rabbit psoas myofibrils are similar or even exceed the active forces (Bartoo et al., 1997; Joumaa et al., 2007). With increasing passive force and decreasing active force contributions at increasing sarcomere lengths, the structures determining sarcomere length non-uniformities in the active myofibril also change from a dominance of the active structures at the short testing length, to a dominance of the passive structures at the long testing length.

In single myofibrils, the exclusive provider of passive force is titin (e.g., Bartoo et al., 1997). Therefore, it is reasonable to propose the hypothesis that the sarcomere length non-uniformities in passive myofibrils are largely (maybe even exclusively) caused by the non-uniformities either in the number or the stiffness of titin filaments in adjacent sarcomeres. Although not systematically measured, sarcomere diameters (and thus presumably cross-sectional areas) of sarcomeres in myofibrils tend to differ, suggesting that the serially arranged sarcomeres may have different amounts of contractile proteins and titin filaments. A small (diameter/area) sarcomere comprised of fewer titin filaments thus would be expected to be longer, and individual titin forces greater, than a large (diameter/area) sarcomere that contains more titin filaments than the small sarcomere. Also, rabbit psoas titin isoforms are of two distinct types with different stiffness (Neagoe et al., 2003; Prado et al., 2005). If the distribution of these two isoforms differs between sarcomeres, the average stiffness of titin may differ as well, thus the sarcomere with the “softer” average titin isoform would be stretched to a longer sarcomere length than a sarcomere with “greater” average titin stiffness, thus accounting for the sarcomere length non-uniformities. Finally, titin filaments have a non-linear stiffness, and the stiffness of titin at a given sarcomere length can differ substantially depending on the amount of unfolding of segments in the PEVK and immunoglobulin regions (Linke et al., 1996; Kellermayer et al., 1998; Eckels et al., 2018). Differences in segment unfolding of titin in one sarcomere compared to the next could be due to the random nature of protein folding/unfolding or could be achieved by differences in the short-term history of sarcomere elongation caused, for example, by an actively stretched muscle/myofibril. If a sarcomere during a dynamic contraction was stretched more than another sarcomere, and thus, more protein unfolding would have taken place, it might take minutes, before a comparable resting state between the two sarcomeres might be achieved (Herzog et al., 2012; Herzog et al., 2014).

In activated myofibrils, sarcomere length non-uniformities are likely governed by the amount of active force that allows for a force equilibrium between neighboring sarcomeres of a myofibril. As discussed above, sarcomeres in myofibrils have different diameters, and thus presumably different amounts of contractile proteins and different strength capacities for identical contractile conditions. We observed that activation of myofibrils in our tests resulted in a dispersion of sarcomeres around some mean length of about 3.4 µm. Sarcomeres longer than 3.4 µm in the passive state tended to become longer with activation, and sarcomeres shorter than 3.4 µm tended to become shorter (Figure 5). This result makes sense because the testing lengths used in this study (2.7–3.6 µm) were on the descending limb of the force-length relationship for rabbit psoas muscles (Herzog et al., 1992). Therefore, increasing sarcomere lengths is associated with a decrease in actin-myosin filament overlap, and thus a decrease in the capacity to produce active force (Huxley, 1957; Gordon et al., 1966; Huxley, 1969). Therefore, the following scenario seems feasible to explain the dispersion of sarcomere lengths upon activation in our experiments.

(i) Sarcomeres have non-uniform length in the passive state, with small diameter sarcomeres likely being longer than large diameter sarcomeres because of a smaller number of titin filaments.
(ii) Upon activation, the small diameter passively long sarcomeres also have less contractile proteins than the large diameter passively short sarcomeres, and the overlap in actin-myosin filament is reduced, both factors contributing to a reduced capacity for active force production.
(iii) For the reasons mentioned in (ii), the passively long sarcomeres are stretched further upon activation, while the passively short sarcomeres shorten.
(iv) In activated myofibrils, stretching of the sarcomeres increases the force due to the increased average cross-bridge force, the increased contribution of titin to force, and the residual force enhancement property, while shortening of activated sarcomeres decreases the force due to a reduction in the average cross-bridge force, the decreased contribution of titin to force, and the residual force depression property of muscles, thus leading towards sarcomere force equilibrium (Abbott and Aubert, 1952; Maréchal and Plaghki, 1979; Edman and Tsuchiya, 1996; Herzog and Leonard, 1997; Lee and Herzog, 2002; Joumaa et al., 2007; Herzog et al., 2010; Rassier and Pavlov, 2012; Herzog, 2014).

In a muscle, in contrast to myofibrils, sarcomeres are arranged in series and in parallel. Despite these additional parallel attachments, sarcomere length non-uniformities in muscles and fibres are similar to those observed here in single myofibril preparations (e.g., Huxley and Peachey, 1961; Llewellyn et al., 2008; Moo et al., 2016). It is not clear what functional implications sarcomere length non-uniformities have in muscles, but the force-length relationship appears unaffected and can be represented well with the mean sarcomere length in intact frog tibialis anterior muscle (Moo et al., 2017), despite great sarcomere length non-uniformities. Some muscles, like indirect flight muscles of insects, have virtually perfect uniform sarcomere lengths, but this uniformity seems to come at the expense of great passive force and a small physiological excursion. Maybe sarcomere length non-uniformity allows for great sarcomere excursion at the expense of small passive force, and thus, great functional capacity and diversity. The parallel arrangement of myofibrils in entire muscle enables muscles to contract in a controlled and synchronized manner, facilitating movements ranging from fine motor skills to powerful locomotion. The study of isolated myofibrils provides valuable insights into the fundamental properties of sarcomeres, but it is important to acknowledge that the *in vivo* muscle architecture, with sarcomeres arranged in series and in parallel, plays a crucial role in achieving the diverse and intricate functions of skeletal muscles.

There are limitations that need to be considered when interpreting the findings of this study. First, forces were not measured with a compliant cantilever as we have often done previously, because we used stiff cantilevers and bonded them to avoid excessive compliance of the measurement system upon activation and deactivation of the myofibrils. However, force was not deemed a relevant factor when exploring sarcomere length nonuniformities in active and passive myofibrils, especially since all myofibrils were fully activated, thus producing maximal forces. Another limitation was that we did not measure individual sarcomere diameters and cross-sectional areas. Myofibrils and sarcomeres have on average a diameter of about 0.7 µm (or 700 nm) for a cross-sectional area (assuming a cylindrical shape, which is not guaranteed) of about 1.5 µm^2^. With the resolution of light microscopy (about 200 nm) and a reliable accuracy of about 100 nm, differences in sarcomere diameter/cross-sectional area were deemed to have too great an error margin to be used reliably. Furthermore, if sarcomere cross-sectional areas were measured in future studies, it would be important to measure them at a consistent sarcomere length (for example, the optimal sarcomere length for the given species), as it is well known that lattice spacing in sarcomeres, and thus sarcomere diameter and/or cross-sectional area, obey the law of constant volume, as first shown by HE Huxley in his 1952 dissertation at the university of Cambridge, and later confirmed many times. Therefore, comparing, for example, two sarcomeres of equal volume but at 2.0 and 2.8 µm in length, one would expect the short sarcomere to have a diameter of about 18% greater than the diameter of the long sarcomere. In order to support some of the presented ideas regarding the origin of the observed sarcomere length non-uniformities, a careful and accurate measurement of sarcomere cross-sectional area at a given sarcomere length, or better yet, a determination of the number of contractile proteins in the serial sarcomeres of a myofibril, would be ideal and needs to be done in the future.

Sarcomere length non-uniformities did not occur randomly but were mostly repeatable for cyclically induced active and passive conditions. We propose that in passive myofibrils, sarcomere length non-uniformities are likely due to differing numbers of titin and the different stiffnesses of titin in serially arranged sarcomeres. Activation causes a dispersion of sarcomere length non-uniformities with passively long sarcomeres tending to become longer and passively short sarcomeres tending to become shorter. This dispersion is likely caused by different amounts of contractile proteins between serially arranged sarcomeres and the properties of the descending limb of the force-length relationship at which these tests were conducted.

## Data Availability

The original contributions presented in the study are included in the article/Supplementary material, further inquiries can be directed to the corresponding author.
