# Supplementary figures and images for: Gaining new understanding of sarcomere length non-uniformities in skeletal muscles
Source: Front Physiol. 2024 Jan 11;14:1242177. doi: 10.3389/fphys.2023.1242177 (PMC10808998; doi:10.3389/fphys.2023.1242177)

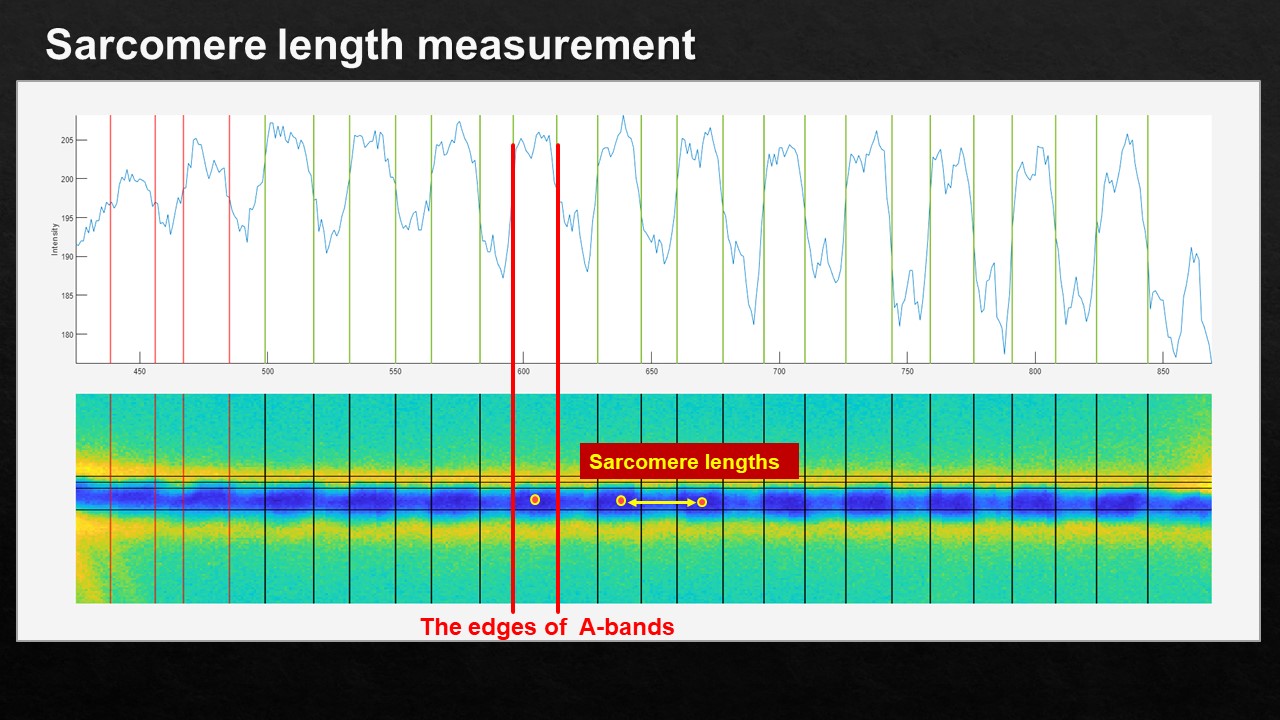

Supplement: Supplementary file 2 [file Image1.JPEG]
