# Supplementary material for: Gaining new understanding of sarcomere length non-uniformities in skeletal muscles
Source: Front Physiol. 2024 Jan 11;14:1242177. doi: 10.3389/fphys.2023.1242177 (PMC10808998; doi:10.3389/fphys.2023.1242177)
Supplement: Supplementary file 3 [file DataSheet1.PDF]

Table 1. Individual myofibril data in each passive and active state

Myofibril (µm)	n	P_0	P_1	P_2	P_3	A_1	A_2	A_3
**SL avg 2.7µm**								
1	22	2.89±0.13	2.80±0.15	2.78±0.18	2.73±0.30	2.64±0.37	2.61±0.43	2.56±0.58
2	26	2.49±0.12	2.44±0.08	2.43±0.08	2.44±0.10	2.35±0.56	2.30±0.16	2.27±0.80
3	26	2.85±0.06	2.77±0.08	2.76±0.08	2.71±0.12	2.62±0.38	2.54±0.50	2.52±0.47
4	15	2.76±0.06	2.70±0.04	2.70±0.06	2.62±0.13	2.45±0.48	2.48±0.39	2.46±0.47
5	19	2.63±0.09	2.53±0.16	2.49±0.15	2.42±0.20	2.33±0.41	2.38±0.30	2.41±0.16
6	25	2.69±0.05	2.64±0.10	2.52±0.15	2.50±0.16	2.26±0.36	2.35±0.39	2.41±0.34
7	23	2.59±0.15	2.59±0.17	2.54±0.24	2.53±0.30	2.49±0.72	2.55±0.58	2.51±0.54
**Total/Mean**	**156**	**2.69±0.18**	**2.64±0.17**	**2.60±0.20**	**2.57±0.24**	**2.45±0.50**	**2.46±0.49**	**2.44±0.53**
**SL avg 3.2µm**								
8	24	3.28±0.11	3.23±0.15	3.28±0.18	3.22±0.22	3.20±0.35	3.17±0.33	3.23±0.47
9	24	3.34±0.17	3.27±0.21	3.23±0.20	3.25±0.23	3.32±0.37	3.22±0.39	3.18±0.33
10	20	3.28±0.14	3.25±0.24	3.25±0.32	3.25±0.34	3.25±0.59	3.20±0.87	3.21±0.77
11	16	3.24±0.17	3.26±0.35	3.31±0.29	3.31±0.38	3.10±0.47	3.20±0.61	3.21±0.51
12	25	3.06±0.10	3.04±0.10	3.02±0.14	3.01±0.19	2.93±0.40	3.02±0.23	3.05±0.28
13	12	3.12±0.14	3.03±0.18	3.09±0.18	3.13±0.26	2.85±0.52	2.88±0.32	2.98±0.45
**Total/Mean**	**121**	**3.22±0.17**	**3.19±0.23**	**3.20±0.25**	**3.19±0.29**	**3.13±0.47**	**3.13±0.51**	**3.15±0.49**
**SL avg 3.6µm**								
14	17	3.54±0.21	3.52±0.29	3.53±0.42	3.46±0.34	3.52±0.70	3.44±0.62	3.41±0.69
15	18	3.70±0.26	3.69±0.33	3.74±0.35	3.81±0.32	3.67±0.53	3.64±0.57	3.73±0.46
16	13	3.65±0.23	3.67±0.44	3.68±0.46	3.66±0.40	3.75±0.49	3.72±0.79	3.74±0.87
17	19	3.72±0.23	3.67±0.28	3.69±0.28	3.66±0.29	3.67±0.42	3.60±0.52	3.59±0.56
18	18	3.33±0.15	3.38±0.31	3.38±0.31	3.40±0.31	3.40±0.47	3.44±0.37	3.41±0.38
19	23	3.57±0.20	3.62±0.25	3.62±0.28	3.65±0.33	3.64±0.51	3.73±0.63	3.67±0.58
20	27	3.45±0.14	3.59±0.28	3.53±0.31	3.61±0.33	3.34±0.68	3.56±0.77	3.49±0.60
**Total/Mean**	**135**	**3.56±0.27**	**3.59±0.35**	**3.59±0.36**	**3.61±0.37**	**3.55±0.58**	**3.59±0.64**	**3.57±0.61**
