# Supplementary material for: Gaining new understanding of sarcomere length non-uniformities in skeletal muscles
Source: Front Physiol. 2024 Jan 11;14:1242177. doi: 10.3389/fphys.2023.1242177 (PMC10808998; doi:10.3389/fphys.2023.1242177)
Supplement: Supplementary file 4 [file DataSheet2.docx]

Supplementary Material

Gaining New Understanding of Sarcomere Length Non-uniformities in Skeletal Muscles

Meng Li*, TR Leonard, SW Han, EK Moo

*** Correspondence:** Walter Herzog: wherzog@ucalgary.ca

# Supplementary Data

The dataset has been uploaded as one of the supplementary materials.

# Supplementary Figures and Tables

## Supplementary Figures


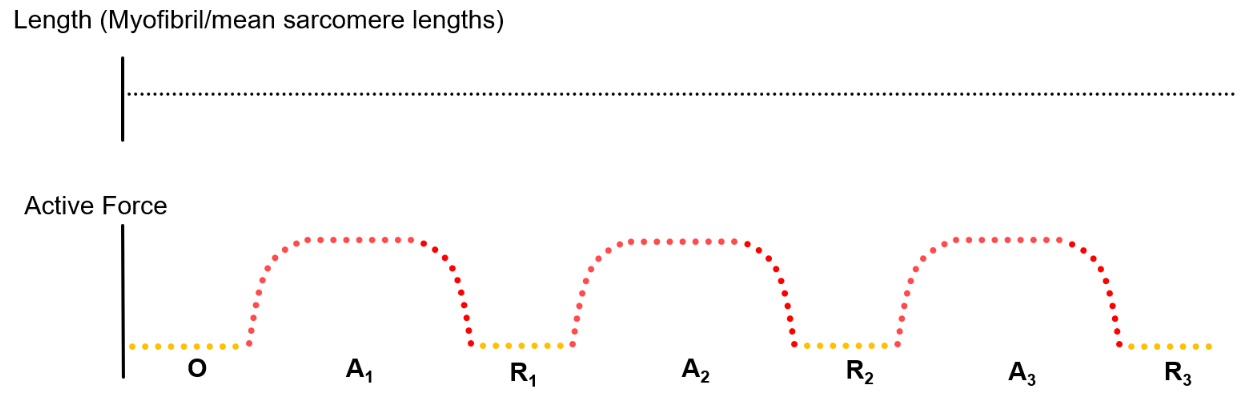


**Supplementary Figure 1.** Experimental Protocol: Sarcomere lengths were measured at seven different instances during the experimental protocol that involved three activation-deactivation cycles. The total myofibril length remained unchanged throughout these cycles. Yellow dots indicate the relaxed states with sarcomere length measurements made prior to activation (0) and following the three activation-deactivation cycles (R1, R2, and R3, respectively); Red dots indicate the active states with measurements made during the three activation periods (A1, A2, and A3, respectively), once steady state forces were reached.

**Supplementary Figure 2.** Activation (going from the relaxed to the activated state) produced an increase in sarcomere lengths non-uniformities at each average sarcomere length that was tested but repeat activation/deactivation did not change sarcomere length non-uniformity in the passive conditions (0 to R3) or the active conditions (A1 to A3). ⅰ: Sarcomere length non-uniformity expressed as mean standard deviations. ⅱ: Sarcomere length non-uniformity expressed as mean coefficients of variation. The absolute sarcomere length non-uniformities were greatest for the longest average sarcomere lengths (3.6µm) tested (ⅰ), but the coefficients of variation were greatest for the shortest (2.7µm) test group of activated myofibrils (ⅱ).

**SLs during reference passive state O (µm)**

**Ave SL: 2.7 µm Ave SL: 3.2 µm Ave SL: 3.6 µm Sarcomeres outside the ±0.2µm reliability boundaries**

**Supplementary Figure 3.** Sarcomere lengths of the original passive state (0 = before the first activation) vs. sarcomere lengths in the passive/relaxed state after the first, second and third activation/deactivation cycles (R1 – Figure ⅰ; R2 – Figure ⅱ; and R3 – Figure ⅲ, respectively). Red squares are sarcomeres from the short group (nominal average sarcomere lengths of about 2.7 µm). Blue squares are from the middle group (average sarcomere length of about 3.2 µm). Green squares are from the long group (average sarcomere length of about 3.6 µm). Yellow squares are sarcomeres whose length changes are greater than the reliability of measurement of ± 0.2 µm. The solid line represents the best fitting linear regression that is forced through the origin (0/0) of the coordinate system. The dotted lines are offset from the best fitting regression line by ±0.2 µm, which represents the day-to-day reliability of sarcomere length measurements. In other words, sarcomeres within these lines are considered to be of the same length.

**Ave SL: 2.7 µm Ave SL: 3.2 µm Ave SL: 3.6 µm Sarcomeres outside the ±0.2µm reliability boundaries**

**Supplementary Figure 4.** Sarcomere lengths of the original active state (A1 = first activation) vs. sarcomere lengths in the active state following the second and third activation/deactivation cycles (A2 – Figureⅰ; A3 – Figure ⅱ, respectively). Red circles are sarcomeres from the short group (nominal initial average sarcomere lengths of about 2.7 µm). Blue circles are from the middle group (average initial sarcomere length of about 3.2 µm). Green circles are from the long group (average initial sarcomere length of about 3.6 µm). Yellow circles are sarcomeres whose length changes are greater than the measurement of day-to-day reliability of ±0.2 µm. The solid line represents the best fitting linear regression that was forced through the origin (0/0) of the coordinate system. The dotted lines are offset from the best fitting regression line by ±0.2 µm, which represents the day-to-day reliability of sarcomere length measurements. In other words, sarcomeres within these lines are considered to be of the same length.

**＋ × △ Ave SL: 2.7 µm ＋ × △ Ave SL: 3.2 µm ＋ × △ Ave SL: 3.6 µm**

**Linear (Avg SL: 2.7 µm) Linear (Avg SL: 3.2 µm) Linear (Avg SL: 3.6 µm)**

**Supplementary Figure 5.** Sarcomere lengths of the original passive state (0 = before activation) vs. sarcomere lengths in the active state of the first activation (A1 – Figureⅰ). Sarcomere lengths of the passive state (R1 = after the first activation/deactivation cycle) vs. sarcomere lengths in the active state of the second activation (A2 – Figure ⅱ). Sarcomere lengths of the passive state (R2 = after the second activation/deactivation cycle) vs. sarcomere lengths in the active state of the third activation (A3 – Figure ⅲ). Red symbols are sarcomeres from the short group (nominal average sarcomere lengths of about 2.7 µm). Blue symbols are from the middle group (average sarcomere length of about 3.2 µm). Green symbols are from the long group (average sarcomere length of about 3.6 µm). The solid lines of red, blue, green represent the best fitting linear regression for the short group, middle group, and long group myofibrils respectively.


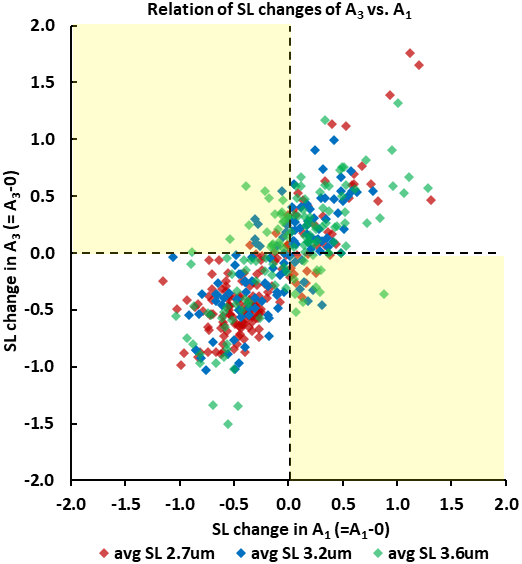


**0.72**

**0.45**

**0.32**

**0.19**

**0.39**

**0.43**

**0.07**

**0.07**

**0.08**

**0.02**

**0.09**

**0.17**


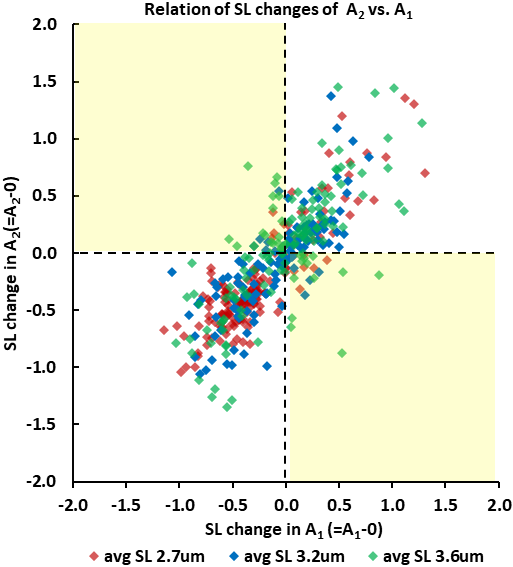


**0.71**

**0.48**

**0.34**

**0.20**

**0.39**

**0.42**

**0.06**

**0.07**

**0.09**

**0.03**

**0.07**

**0.15**

**ⅰ**

**ⅱ**

**Supplementary Figure 6.** (ⅰ) Sarcomere length changes in the second activation cycle (going from the initial relaxed state “0” to the second active state “A2”) as a function of the sarcomere length changes occurring during the fist activation cycle (going from the initial relaxed state “0” to the first active state “A1”), and (ⅱ) the corresponding sarcomere length changes going from the initial relaxed state “0” to the third active state “A3” as a function of the length change in the first activation cycle (going from “0” to “A1”). Data points in the first and third quadrant indicate that sarcomeres were shortening/elongating in both activation cycles, while data points in the second and fourth quadrant indicate that sarcomeres were elongating/shortening or shortening/elongating in the first and subsequent activation cycle. The proportion of sarcomeres falling into the four quadrants are indicated in the red, blue, and green numbers for the short (2.7 µm), middle (3.2 µm) and long (3.6 µm) average sarcomere length tests. The vertical and horizontal lines through the zero values indicate zero length change of a sarcomere in the activation cycle going from the relaxed/passive to the active state. The corresponding vertical and horizontal lines at ±0.2 µm from the zero lines indicate the reliability of our sarcomere length measurements. In other words, sarcomere length changes within the ± 0.2 µm lines are considered zero length change, or length changes that cannot be said to be greater than zero with a degree if certainty.
